# Habitat suitability and ecological niche profile of major malaria vectors in Cameroon

**DOI:** 10.1186/1475-2875-8-307

**Published:** 2009-12-23

**Authors:** Diego Ayala, Carlo Costantini, Kenji Ose, Guy C Kamdem, Christophe Antonio-Nkondjio, Jean-Pierre Agbor, Parfait Awono-Ambene, Didier Fontenille, Frédéric Simard

**Affiliations:** 1 UR016 CCPV, IRD, BP 64 501, Montpellier, France; 2 Laboratoire de Recherche sur le Paludisme, OCEAC, Yaoundé, Cameroon; 3 US140 ESPACE, IRD, Montpellier, France; 4 IRSS, Bobo Dioulasso, Burkina Faso

## Abstract

**Background:**

Suitability of environmental conditions determines a species distribution in space and time. Understanding and modelling the ecological niche of mosquito disease vectors can, therefore, be a powerful predictor of the risk of exposure to the pathogens they transmit. In Africa, five anophelines are responsible for over 95% of total malaria transmission. However, detailed knowledge of the geographic distribution and ecological requirements of these species is to date still inadequate.

**Methods:**

Indoor-resting mosquitoes were sampled from 386 villages covering the full range of ecological settings available in Cameroon, Central Africa. Using a predictive species distribution modeling approach based only on presence records, habitat suitability maps were constructed for the five major malaria vectors *Anopheles gambiae, Anopheles funestus, Anopheles arabiensis, Anopheles nili *and *Anopheles moucheti*. The influence of 17 climatic, topographic, and land use variables on mosquito geographic distribution was assessed by multivariate regression and ordination techniques.

**Results:**

Twenty-four anopheline species were collected, of which 17 are known to transmit malaria in Africa. Ecological Niche Factor Analysis, Habitat Suitability modeling and Canonical Correspondence Analysis revealed marked differences among the five major malaria vector species, both in terms of ecological requirements and niche breadth. Eco-geographical variables (EGVs) related to human activity had the highest impact on habitat suitability for the five major malaria vectors, with areas of low population density being of marginal or unsuitable habitat quality. Sunlight exposure, rainfall, evapo-transpiration, relative humidity, and wind speed were among the most discriminative EGVs separating "forest" from "savanna" species.

**Conclusions:**

The distribution of major malaria vectors in Cameroon is strongly affected by the impact of humans on the environment, with variables related to proximity to human settings being among the best predictors of habitat suitability. The ecologically more tolerant species *An. gambiae *and *An. funestus *were recorded in a wide range of eco-climatic settings. The other three major vectors, *An. arabiensis*, *An. moucheti*, and *An. nili*, were more specialized. Ecological niche and species distribution modelling should help improve malaria vector control interventions by targeting places and times where the impact on vector populations and disease transmission can be optimized.

## Background

The interactions between a species and its environment are reflected in the distribution of its abundance in both space and time [1]. Species are expected to be non-randomly distributed across different ecological settings, as a result of their specific ecological requirements and tolerance towards deviations from their optimal conditions [2,3]. Predictions of species geographic distributions can be based upon mathematical models relating field observations of occurrences to a set of environmental variables [4,5]. This kind of approach has been used to explore ecological niche requirements and to predict the potential distribution of a focal species [6]. Such predictions can be used to tackle a wide range of issues such as conservation of biodiversity, the management of species of economic interest, or evaluation of the risks linked with biological invasions [7-10]. Species distribution models are also gaining interest as a tool to evaluate and/or predict the risk of exposure to infectious diseases and their vectors, such as malaria [11-14], Chagas disease [15] or dengue [16]. Risk maps have been produced by correlating geo-referenced epidemiological and environmental data to describe, explain and predict malaria risk at localities where epidemiological data are not available [11,17,18]. Mosquito life-history traits, such as growth rates and survival and the duration of the sporogonic cycle of *Plasmodium *in its vector, are strongly dependent upon temperature and moisture conditions on the ground. Thus, eco-climatic profiles inferred from remotely sensed images can be used as predictors of mosquito distribution patterns and average levels of transmission of malaria parasites by these vectors [12].

Malaria transmission dynamic is highly variable throughout Africa. These variations mirror, at least to some extent, the great heterogeneity of eco-climatic settings present across sub-Saharan Africa [19]. In this continent, about twenty out of 140 anopheline species have been incriminated in malaria transmission [20,21]. However, only five species are responsible for more than 95% of the overall transmission, and are therefore considered the major malaria vectors in Africa: *Anopheles gambiae*, *Anopheles arabiensis*, *Anopheles funestus*, *Anopheles moucheti*, and *Anopheles nili *[19,21]. The remaining 5% is due to "secondary" malaria vectors of local importance. Differences in ecological requirements, longevity and feeding behaviour (e.g. anthropophily) account for the different roles played by major and secondary vectors in malaria transmission [22]. Whereas variations in longevity and anthropophily within and between vectors species have been documented under a wide range of settings, qualitative and quantitative assessments of species' ecological requirements are still few, even for major vector species [19,23].

This paper focuses on the determination of ecological requirements for malaria vectors in Cameroon, a country in Central Africa covering a wide range of ecological and climatic domains. This great environmental heterogeneity increases the diversity of the malaria transmission system, with as much as 48 anophelines species reported [24-26], among which 17 have been found infected with human malaria parasites [22,27-30]. Geographical Information Systems and Ecological Niche Factor Analysis (ENFA) [3] were employed to build predictive habitat suitability maps for the five major malaria vectors on a country-wide scale, and to compare their respective ecological requirements and niche parameters. In addition, the ecological habitat profiles for the 10 most common anopheline species found in Cameroon were described using Canonical Correspondence Analysis (CCA). This study improves current knowledge of malaria vector distribution in Cameroon and highlights relevant similarities and differences in ecological requirements of the different mosquito species present in this area. The relevance of these findings for malaria vector control in Africa is discussed.

## Methods

### Study area and mosquito sampling

**Figure 1:**
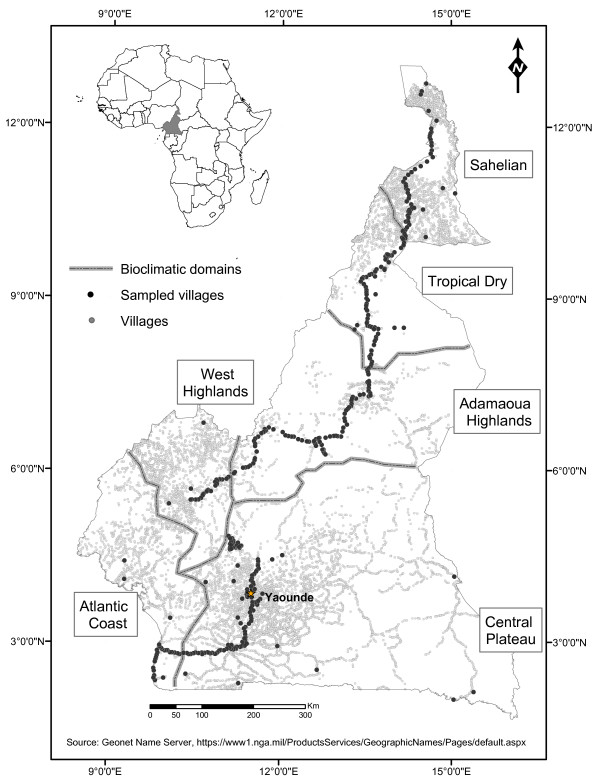
**Topographic map of Cameroon**. Localities sampled for the mosquito domestic fauna are shown as dark dots (N = 386) among all the recorded populated places present across Cameroon shown as gray dots (N = 10,700). Dotted lines delimit the main bio-geographic domains [31].

Mosquitoes were collected across a wide range of geographical, ecological and climatic conditions that occur throughout Cameroon (Figure 1). This central African country covers an area of approximately 475,000 km^2 ^between 2-12° latitude North and 8-16° longitude East. It is characterized by several bio-geographic domains, from arid savannas in the north to the equatorial rainforest in the south. Highlands in the central and western part of the country contribute to increase the diversity of ecological settings and seasonality [31]. Mosquito presence was recorded from previously published data and *ad hoc *surveys covering 386 villages spread across all the country's eco-geographical settings (Figure 1): 32 villages were surveyed between 1998 and 2003 [22], and 354 villages were surveyed in 2005-2007. The 2005 survey sampling plan, which covered 305 localities, was conceived to cover all the ecological and climatic settings occurring across the country, with sampled sites selected at random from the most comprehensive list of populated places available for Cameroon at the time (Figure 1--further details are given in [28]). Mosquitoes were collected while resting inside human dwellings by indoor pyrethroid insecticide spraying in an average number of 10 rooms per village (for further details, see [22,32]). Mosquito species were identified upon collection using morphological identification keys [33,34]. Field specimens were stored individually in tubes containing a desiccant and kept at -20°C until processed in the laboratory. Molecular diagnostic tools were used to identify species within species complexes [30,35-39].

### Environmental data

The study area was modelled as a raster map (1 km^2 ^per pixel) overlaid on the UTM Coordinate System (Zone 33N and 32N for Cameroon). Four classes of environmental descriptors, totalling 17 eco-geographical variables (EGVs), were used in the analysis:

(i) topographic variables [source: Shuttle Radar Topography Mission-SRTM [40], including elevation (in meters), slope (derived from elevation data), aspect (derived from slope data) and hydrographic network (computed as a quantitative raster layer, attributing to each pixel its minimum distance to a water body). Each layer was formatted in a quantitative raster format. This was achieved using the *Spatial Analyst *extension in *ArcGIS v8.3 *with a number of customized scripts and downloads for geoprocessing available from the ESRI websites [41,42] and the software *Erdas Imagine 8.0 *for resampling of corrected/updated data at the appropriate resolution (i.e., 1 km^2 ^cells).

(ii) climatic variables [source: *LocClim *database developed by the Food Agriculture Organization - FAO [43], including rainfall (in mm), temperature (in °C), evapo-transpiration (in mm), relative humidity (water vapour pressure in %), mean number of hours of sunlight per day (hours), and wind speed (in m.s^-1^). These data were yearly means, averaged over the past 30 years. The data from individual field weather stations were interpolated using an inverse distance weighting algorithm in the *Spatial Analyst *module of ArcGIS v. 8.3;

(iii) habitat variables [source: *Global Land Cover 2000 Project *[44]. This database embodied 22 land cover types, however due to computational constraints, the number of variables entered in the analysis was reduced to 5 classes of land cover: dense evergreen forest, deciduous woodland, forest/savannas mosaic, dry savannas and cropland. Each land cover type constituted a separate layer that was encoded in Boolean format;

(iv) variables related to human population pressure on the environment [Geonet Name Server [45], updated for Cameroon by the Dyepca Project developed by the US140 of IRD in Montpellier, France] including minimum distance to localities and minimum distance to the main road network.

The distribution of each EGV was normalized by the Box-Cox algorithm [46]. All layers were smoothed using the median index calculated over the 10 × 10 neighbouring spatial units of a focal cell [3,47] and exported as *Idrisi *files [48] into the software *Biomapper 4.0 *[49] to perform an Ecological Niche Factor Analysis (ENFA).

### Ecological niche factor analysis

The ENFA is a species distribution model based on the ecological niche concept developed by Hutchinson [2] in a multivariate statistical framework. This model has been described in detail by [3,4] and [50]. The ENFA uses only data on the presence of the target species, and requires a raster map describing species occurrence, encoded in Boolean format. Villages where the focal species was observed were given the value of 1, while villages where the species was not found were encoded as 0; the latter are not taken into account by the ENFA. The ENFA summarizes the overall information by two indices. The first is termed 'marginality': it maximizes the multivariate distance of EGVs between the cells occupied by the species and the cells within the whole reference area (e.g. the whole of Cameroon). This index provides information about the extent to which the species niche optimum departs from the most frequent set of eco-geographical conditions in the spatial multivariate reference set. Global marginality close to one means that the species lives in a particular habitat relative to the reference area. Conversely, a value of zero indicates that the species is found everywhere [3]. The second and subsequent factors are termed 'specialization' factors: they account for the decreasing residual variance after removal of upper-ranked explanatory factors, and denote the extent of the species EGVs distribution width with respect to the overall distribution of the EGVs in the whole reference area. The inverse of specialization is therefore a measure of the species tolerance to conditions that are increasingly different from its optimum [3,9]. A global tolerance of one indicates no specialization at all; any value below one indicates some form of specialization. Marginality and specialization are uncorrelated factors, with the bulk of the information contained within the first factors [3].

### Habitat suitability maps

Habitat suitability (HS) maps represent the variation in space of the likelihood of occurrence of a focal species. HS maps are based on the calculation of a Habitat Suitability Index (HSI). The HSI was calculated using the median algorithm based on the first significant factors obtained by the ENFA [3,4,50]. The number of significant factors included in the analysis results form the comparison of each factors' eigenvalues to a MacArthur's broken-stick distribution [3,9]. On one factor axis, the HSI calculation is based on a count of cells from the species distribution that lay at least as far apart from the median as the focal cell. This procedure is repeated for each factor included in the HSI calculation. The HSI varies from 0 (worst habitat) to 100 (best habitat), and indicates how the environmental conditions of a spatial unit expressed as a linear combination of the EGVs suit the requirements of the focal species. However, as noted by Hirzel [4], maps produced through the use of a continuous HSI are often spurious and misleading. Reclassified maps using only a few classes of HS are likely to be more accurate in regards to their actual informative content [9]. The number and boundaries of HS classes were defined following Hirzel *et al*, through a detailed inspection of the predicted-to-expected frequency curves (P/E, Boyce's area-adjusted frequencies) obtained for each mosquito species, as implemented in *Biomapper *4.0 [49]. Four classes of HS were defined: unsuitable, marginal, suitable and optimal habitat, according to the best bin combinations of the Boyce Index *B4 *(see below). The boundaries between each class were set as follows: habitat suitability with no presence points (P/E = 0) denotes unsuitable habitat; habitat suitability values for which presences are less frequent than expected by chance alone (0<P/E<1) define marginal habitat; suitable and optimal habitat shared habitat suitability values for which presences are more frequent than expected by chance (P/E>1), the boundary being placed so as to maximize the P/E difference between them and limit overlap in P/E values.

The HS model's predictive power and accuracy was evaluated by means of a Jackknifed 10-fold cross-validation procedure [51] available in *Biomapper 4.0 *[49]. Briefly, the species locations are randomly partitioned into k = 10 mutually exclusive sets. k-1 partitions are be used to compute a HS model and the remaining partition is used to validate it on independent data. This process is repeated k times, each time by leaving out a different partition. This results in k more-or-less different HS maps. By comparing these maps and how they fluctuate, one can assess their predictive power. By default in *Biomapper 4.0*, the partitions are set so that they do not overlap geographically. This makes the cross-validation more robust to spatial auto-correlation (e.g. conspecific attraction). Three model evaluation indices were calculated for each replicate and were characterized by their mean and standard deviation across replicates. The Absolute Validation Index (0 = AVI = 1) is the proportion of presence points in the evaluation partition falling in spatial units with a HSI>50. The AVI indicates how well the model discriminates high-suitability from low-suitability areas [9,47,52]. The Contrast Validation Index (0 = CVI = AVI) is the difference between the AVI of a replicate and the AVI of a null model that would predict habitat suitability at random. The CVI indicates how much the model differs from a random model of habitat suitability. These two measures determine how good the model is at discriminating between suitable and unsuitable habitats for the focal species, but they depend on the choice of an arbitrary threshold (in this case, HSI = 50). The threshold-independent Boyce indices [4,53] were used to provide a more continuous and more reliable measure of the accuracy of the model's predictions. The continuous Boyce index, calculated through the use of a 'sliding window' across the range of HSI values, measures the monotonic increase of the P/E frequency ratio with increasing habitat suitability and is computed by the Spearman's rank correlation coefficient between P/E and HSI. This index varies from -1 to 1, with 0 indicating a random model [4,53]. Similarly, the sequential Boyce index *B4 *was used to assess the predictive power and robustness of the reclassified maps (cf. above), and was computed as the Spearman's rank correlation coefficient between P/E and mean HSI value for each of the four HSI classes defined above, averaged over 10-fold resampling.

### Ecological niche differentiation across species

In order to compare the ecological requirements of different mosquito species in Cameroon, discriminant analyses were performed between pairs of species' ecological niches. While the ENFA compared the distribution of EGVs where a focal species was present to that of the whole reference area, discriminant analysis compared the distribution of EGVs of two species, with the aim to compute a factor that maximizes the difference between species while minimizing the intra-species variance. As this factor is a linear combination of the EGVs, its coefficients can be interpreted to identify which EGVs contributed the most to discriminate the ecological niche of the two species. Hence, the discriminant function indicates for which variables the two species differ most. These computations are integrated in a module of *Biomapper *4.0 [*49*] and were carried out for each combination of species' pairs among the five major malaria vectors.

### Canonical correspondence analysis

CCA was carried out to explore the relationship between mosquito species and EGVs using *CANOCO *v4.0 [54]. CCA assesses the main pattern of variation in community composition accounted for by the environmental variables and draws a graphical representation of the contribution of each EGV to the global species distribution. This analytical technique allows a quick appraisal of how community composition varies with the environment. As all mosquitoes were collected in human settlements, variables related to the human influence on the environment could bias the analysis. Moreover, distance to water bodies was not informative when the ENFA was performed (see Results). Thus, distances to roads, localities and to water bodies were removed from the set of EGVs fitted by CCA. Species data were encoded as presence (1) or absence (0) for each of the sampled localities. Both values were taken into account in the posterior analysis. Statistical significance of the canonical axes was tested with a Monte Carlo permutation test using 5000 permutations.

## Results

### Anopheline species diversity and distribution

**Figure 2:**
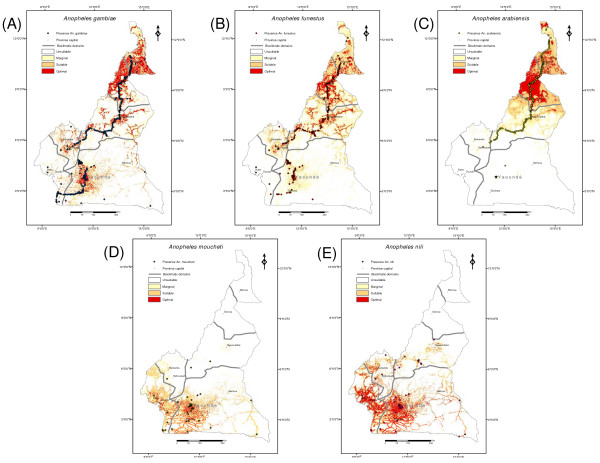
**Habitat suitability maps for the five major malaria vectors in Cameroon**. Dots represent species presence points used for the ENFA: (A) *An. gambiae*; (B) *An. funestus*; (C) *An. arabiensis*; (D) *An. moucheti *and (E) *An. nili*. Different colours identify the four classes of habitat quality.

Twenty-four anopheline mosquito species were recorded during the surveys (Table 1, Figure 2 and see Additional file 1); of these, 17 have been reported as primary or secondary malaria vectors in Africa [22,27,30,55,56]. Some species, among which are all the major malaria vectors, belong to groups or complexes of morphologically similar or identical species [19,34]. In the *An. gambiae *complex, *An. gambiae s.s*. (hereafter *An. gambiae*), *An. arabiensis *and *Anopheles melas *were observed. In the *An. funestus *group, *An. funestus s.s*. (hereafter *An. funestus*), *Anopheles leesoni *and *Anopheles rivolorum- *like were collected. In the *An. nili *group, *An. nili *s.s. (hereafter *An. nili*), *Anopheles carnevalei *and *Anopheles ovengensis *were found in the samples. In the *An. moucheti *group, only *An. moucheti *s.s. (hereafter *An. moucheti*) was detected. Species relative frequencies are given in Table 1. Three species (*An. gambiae*, *An. funestus*, and *An. arabiensis*) were recorded in ≥50% of the sampled villages. Conversely, seven species (*Anopheles squamosus, Anopheles pretoriensis, Anopheles implexus, Anopheles obscurus, An. carnevalei, An. ovengensis *and *Anopheles smithii*) were recorded from a single village each. Only *An. gambiae *and *An. funestus *were widely distributed across all bioclimatic domains throughout the country, whereas most of the other species occurred in a specific domain. For example, *An. arabiensis *was predominant in the northernmost and most arid regions, while *An. moucheti *and *An. nili *were found mainly in the humid forest areas in the South. The greatest species richness was observed in the rainforest, with 18 out of 24 species present.

**Table 1:** Anopheline species recorded in 386 villages from Cameroon

Species	Presence Villages	% of total
An. gambiae*	308	*79.79*
An. funestus*	205	*53.11*
An. arabiensis*	191	*49.48*
An. nili*	38	*9.84*
An. moucheti*	37	*9.59*
An. paludis*	21	*5.44*
An. ziemanni*	15	*3.89*
An. coustani*	12	*3.11*
An. hancocki*	11	*2.85*
An. pharoensis*	11	*2.85*
An. marshallii*	6	*1.55*
An. wellcomei*	6	*1.55*
An. carnevalei*	3	*0.78*
An. leesoni*	3	*0.78*
*An. namibiensis*	3	*0.78*
An. rivulorum-like*	3	*0.78*
An. melas*	2	*0.52*
An. ovengensis*	2	*0.52*
*An. rufipes*	2	*0.52*
*An. implexus*	1	*0.26*
*An. obscurus*	1	*0.26*
*An. pretoriensis*	1	*0.26*
*An. smithii*	1	*0.26*
*An. squamosus*	1	*0.26*

Asterisks identify known malaria vectors.

### ENFA

The accuracy of the ENFA and HS maps depends upon the number and distribution of presence points in the data set [3,50]. It was possible to compute the ENFA only for the five most common malaria vectors in Cameroon: *An. gambiae, An. funestus, An. arabiensis, An. moucheti *and *An. nili*, because the prevalence of other anophelines was too low.

All these five mosquitoes exhibited high global marginality values (>1 in all cases), indicating that they occupied a relatively small portion of the set of environmental conditions (defined by the EGVs) available in Cameroon. *Anopheles gambiae *and *An. funestus *exhibited the lowest global marginality values (1.11 and 1.19, respectively), suggesting both species are more 'generalist' relative to the other three anophelines (Table 2). Global tolerance indices were generally low, ranging from 0.26 in *An. arabiensis *to 0.69 in *An. gambiae*, indicating low tolerance towards deviations from the species optimal conditions. *Anopheles gambiae *and *An. funestus *had the highest values of tolerance (0.69 and 0.64, respectively), indicating that they are more likely than the other species to colonize sub-optimal habitats.

**Table 2:** Contribution of 17 eco-geographical variables to the Marginality and Specialization factors of the ENFA for five major malaria vectors in Cameroon.

	MARGINALITY^1^					SPECIALIZATION^2^				
		
	*Anopheles gambiae *22%	*Anopheles funestus *23%	*Anopheles arabiensis *63%	*Anopheles moucheti *37%	*Anophele s nili *16%	*Anopheles gambiae *21%	*Anopheles funestus *16%	*Anopheles arabiensis *16%	*Anopheles moucheti *40%	*Anopheles nili *41%
Global Values	1.109	1.186	1.763	1.482	1.265	0.691	0.638	0.259	0.291	0.437
Cropland	++	+	++	++	++	0	*	*	0	*
Distance to water bodies	0	0	0	0	0	0	0	0	0	0
Distance to localities	-------	------	----	------	-------	*	0	0	*	*
Distance to roads	-----	----	---	---	----	*	*	0	**	*
Evapotranspiration	+	++	++++	---	--	*	**	*******	*********	*********
Evergreen Forest	--	---	---	++	+	*****	****	*	*	**
Sunlight exposure	+	+	+++	----	---	*****	**	*	***	**
Forest/savannas mosaic	0	0	0	-	+	0	0	0	0	0
Rainfall	0	--	---	++	++	*****	******	0	0	0
Dry savannas	++	++	++	0	0	0	0	0	0	0
Deciduous woodland	+	++	0	0	++	0	*	*	0	0
Temperature	+	+	++	0	-	***	***	*	**	*
Elevation	-	0	-	-	0	*	0	0	*	*
Aspect	0	-	0	0	0	0	0	0	0	0
Slope	-	-	-	-	--	*	*	0	0	*
Wind speed	++	+++	+++	+	+	**	****	*******	*	0
Water vapor pressure	-	--	---	+++	++	*	**	*	0	**

Percentages indicate the amount of total variance explained by each factor.

^1 ^The symbol "+" indicates that the focal species was found in locations with higher values than average for that EGV. The symbol "-" indicates the reverse. The greater the number of symbols, the higher the marginality, with "0" denoting weak marginality.

^2 ^The symbol "*" indicates that the focal species occupied a narrower range of values for the EGV than those available in the reference set (i.e. specialization). The greater the number of symbols, the higher the specialization, with "0" denoting no specialization.

Table 2 shows the coefficients of each EGV on the marginality and first specialization factors for the five most common mosquito species. The marginality factor represents the best combination of all EGVs characterizing the ecological niche of each species at the geographical scale of the spatial reference set. Marginality was particularly important for *An. arabiensis *and explained 63% of the total model variance. The occurrence of all five major malaria vectors was highly positively correlated with variables related to human activity, including proximity to localities, roads, and croplands (Table 2). Further inspection of the coefficients on the marginality factor allows a clear distinction between predominantly 'savanna' species (e.g. *An. gambiae, An. funestus *and *An. arabiensis*), and predominantly 'forest' species (e.g. *An. nili *and *An. moucheti*). In the 'savanna' group, occurrences were positively correlated to increasing evapo-transpiration, sunlight exposure, temperature, and wind speed--whose values are typically higher in dry savannas--and negatively correlated to increased water vapour pressure, rainfall and the presence of rainforest. Exactly the reverse trend was observed for both 'forest' species (Table 2). It is noteworthy, however, that *An. gambiae*, and to a lesser extent *An. funestus*, revealed intermediate values of the coefficients for these EGVs, in agreement with their lower global marginality and higher tolerance indices.

Inspection of the first specialization factor provided further insights on the ecological niche breadth of the five malaria vectors (Table 2). This factor alone explained 41% and 40% of variation in *An. nili *and *An. moucheti*, respectively. Both species showed the highest level of specialization for evapo-transpiration, suggesting this EGV has a major impact in limiting habitat suitability for both species to areas of narrow variations in evapo-transpiration, as in the tropical forest. Moreover, low sunlight exposure, high rainfall and water vapour, and presence of the evergreen forest were correlated with the presence of *An. nili *and *An. moucheti*. *Anopheles arabiensis *showed a lower percentage of explained variation (16%) for this first specialization factor, suggesting that the effect on niche breadth for this species is largely distributed across the other specialization factors. Along this factor, the same trend as in *An. nili *and *An. moucheti *was observed for *An. arabiensis*, although in the opposite direction. In addition, *An. arabiensis *appeared to prefer areas of high wind speed with a high level of specialization for this EGV. Higher wind speed is typically observed in open habitats, which occur at greater frequency in the drier savannas of north Cameroon. *Anopheles gambiae *and *An. funestus *showed similar patterns of specialization, the first factor explaining 21% and 16% of variation in each species, respectively. Both species showed low tolerance towards deviation from their optimal rainfall and temperature conditions while showing strong avoidance for rainforest landscapes. This trend was more pronounced for *An. funestus *than for *An. gambiae*.

### Habitat suitability maps

The number of ENFA factors retained after comparison with a broken-stick distribution to construct Habitat Suitability (HS) maps varied from 2 (explaining 88.5% of total information) for *An. moucheti *to 6 (explaining 88.1% of total information) for *An. funestus*. The median algorithm proposed by Hirzel *et al *was used to derive HS values for *An. gambiae *and *An. funestus *for each cell in the reference area. However, given the high global marginality values observed for *An. arabiensis*, *An. moucheti *and *An. nili*, HS values for these species were derived using the recently proposed area-adjusted median + extremum algorithm, which optimizes the continuous Boyce index for these species (M_ae_, see [50]). Resulting reclassified maps showing four HS classes (optimal, suitable, marginal and unsuitable) are presented in Figure 2 for each of the five most common malaria vectors in Cameroon.

The HS map of *An. gambiae *(Figure 2A) identifies a core favourable habitat in the dry savannas of North Cameroon. Habitat suitability decreases when moving northwards to the most arid regions, and southwards to more humid environments. Patches of favourable habitat, however, were also found in areas where the vegetation cover is degraded by human presence, such as the hilly landscapes in the Adamaoua and Western Highlands, and the densely populated area surrounding Yaounde in the forest domain. The humid Atlantic coast and the sparsely populated areas in the evergreen rainforest of the Congo basin in the East are unsuitable for this mosquito, except in close vicinity to inhabited places. A very similar pattern of HS distribution was observed for *An. funestus *(Figure 2B), although habitat in the rainforest domain appears of overall lower quality for this species. *Anopheles arabiensis*, *An. nili *and *An. moucheti *had a much more restricted distribution of favourable habitat. The reclassified HS map clearly identified the northernmost, xeric regions of Cameroon as the most suitable habitat for *An. arabiensis *(Figure 2C). Patches of suitable habitats were also found at the edge of the Adamaoua and Western Highlands. Further south, across the forested Central plateau and Atlantic belt, the habitat was classified as unsuitable for this species. The HS map for *An. moucheti *(Figure 2D) identified areas of suitable habitat that are substantially more restricted than those of the other species: optimal habitat clustered mainly around Yaoundé, with extensions westwards on the Atlantic coast, southwards and eastwards, in the evergreen rainforest of the Congo basin, along the main networks of roads. Northwards, beyond the evergreen forest distribution limit, the habitat was essentially unsuitable for *An. moucheti*, although patches of marginal and suitable habitat were found in areas where large water reservoirs are found (Adamaoua). Finally, as shown in Figure 2E, the core favorable habitat for *An.nili *was identified in the densely populated areas south and west of the country, within the rainforest and humid savanna regions, with patchy extensions into the highland areas.

HS maps' accuracy was evaluated by means of 10-fold cross-validations for each of the five species (Table 3). The presence-only evaluators AVI and CVI were around 0.5 for every species, indicating that the HS models were able to discriminate between suitable and unsuitable habitats and that the set of EGVs allowed distinguish specific habitats preferred by each species from the overall habitat available in Cameroon. However, high standard deviations indicated rather low robustness. Boyce's indices provided a more continuous assessment of the model and predictive map accuracy. The values of these indices were positive and high for *An. gambiae *and *An. funestus *and somewhat lower for the three other species, especially for *An. moucheti*. Large standard deviation around most estimates reflected low robustness, especially in the case of the continuous model. However, reclassified HS maps were reliable to predict the distribution of HS throughout Cameroon for most species, as indicated by the high and positive values of the Boyce *B4 *index which was maximal in the case of *An. gambiae *and *An. funestus *(Table 3). The lowest *B4 *index, which was associated with a high standard deviation, was that of *An. moucheti *(Table 3), suggesting that the HS map for this species is to be considered provisional at this stage.

**Table 3:** Model evaluation statistics for the habitat suitability maps of five major malaria vectors in Cameroon.

	**AVI^1^**	**CVI^2^**	**Boyce's index continuous^3^**	**Boyce Index B4^4^**
	
*An.gambiae*	0.46 ± 0.13	0.38 ± 0.13	0.67 ± 0.33	1
*An.funestus*	0.50 ± 0.17	0.42 ± 0.16	0.60 ± 0.29	1
*An.arabiensis*	0.43 ± 0.17	0.34 ± 0.17	0.24 ± 0.29	0.88 ± 0.10
*An.moucheti*	0.59 ± 0.39	0.55 ± 0.38	0.28 ± 0.54	0.68 ± 0.54
*An.nili*	0.53 ± 0.32	0.49 ± 0.32	0.21 ± 0.48	0.81 ± 0.24

Higher mean values indicate higher consistency with the evaluation data sets. The lower the standard deviation, the more robust the predictions.

^1 ^AVI varies from 0 to 1; ^2 ^CVI varies from 0 to AVI; ^3 ^Boyce's indices vary from -1 to 1, with 0 indicating a random model.

### Ecological niche comparisons among species

**Figure 3:**
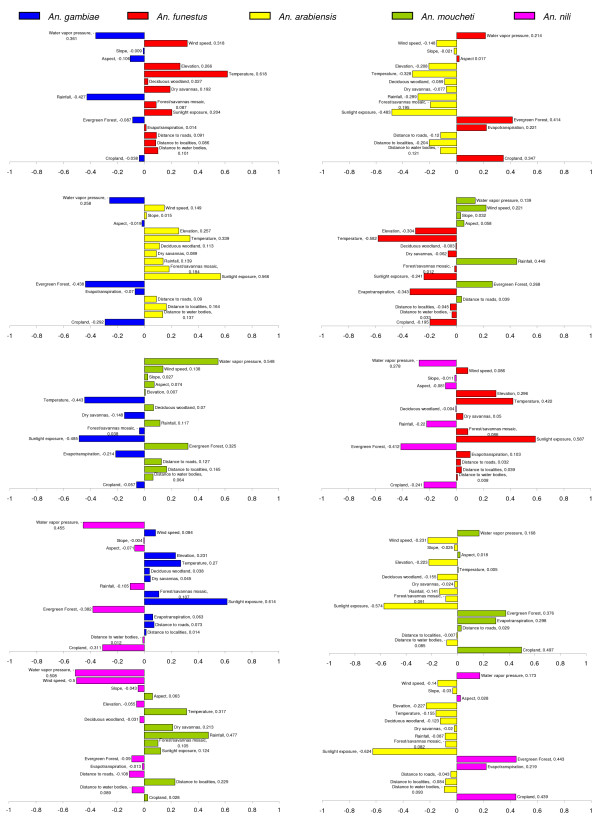
**Coefficients of the discriminant function differentiating the ecological niche of pairs of major malaria vectors based on 17 eco-geographical variables**.

Results of the discriminant analysis of the ecological niches of each species pairs are shown in Figure 3. This kind of analysis indicates for which eco-goegraphical variables the species differ most. The most important differences were observed between 'savanna' and 'forest' species. Not surprisingly, the presence of evergreen rainforest, high water vapour pressure and high rainfall were always associated with 'forest' species. By contrast, sunlight exposure and temperature were always associated with 'savanna' species. These associations can be easily understood on the basis of the divergence in distribution between 'forest' and 'savanna' species at a macrogeographic scale. More subtle inferences can be proposed for pairs of species that tend to occur in the same locales, such as *An. gambiae *and *An. funestus*. In this case, environmental variables spatially discriminating the occurrence of species with similar ecological niches are reflected also in temporal patterns of mosquito population dynamics. For instance, *An. gambiae *occurrence was correlated to higher values of rainfall and water vapour pressure as compared to *An. funestus*. On the other hand, *An. funestus *was associated with higher values of temperature and sunlight exposure (Figure 3). In nature, the peak in abundance of this pair of species is delayed across the rainy season: *An. gambiae *peaks at the climax of the rainy season, when rainfall and humidity are higher and temperature and sunlight lower, whereas the peak of *An. funestus *is further delayed until the beginning of the dry season, when temperature and sunlight are higher and rainfall and humidity lower.

### Canonical correspondence analysis

**Figure 4:**
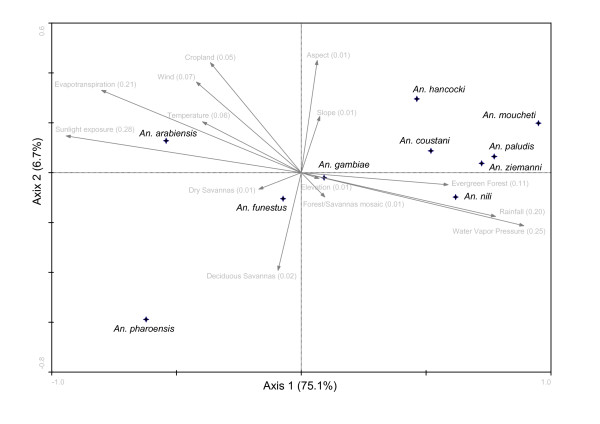
**Ordination biplot diagram showing the dispersion of ten malaria vectors and 14 eco-geographical variables on the first two canonical axes of a Canonical Correspondence Analysis**. Crosses represent the average niche centroid for each mosquito species. In brackets the EGV contribution to total species variance, and the total species variance explained by each canonical axis.

Canonical Correspondence Analysis (CCA, Ter Braak, 1987) was used to analyse the spatial pattern of occurrence of the 10 most common anopheline species in Cameroon: *An. gambiae*, *An. funestus*, *An. arabiensis*, *An. nili*, *An. moucheti*, *Anopheles paludis, Anopheles ziemanni, Anopheles coustani, Anopheles hancocki *and *Anopheles pharoensis*. A graphical representation of the contribution of each EGV to the global species distribution is given in Figure 4, where the first two canonical axes--explaining 81.8% of the total variance in species distribution--are shown. The EGV that contributed most to species distribution was sunlight exposure, which explained 28% of the variance (Figure 4, F-ratio = 59.8, *P *= 0.0007), followed by water vapour pressure, evapo-transpiration and rainfall (25%, 21%, and 20%, respectively). The remaining EGVs had a lower impact on species variation.

The first axis, which explained 75.1% of total species variation, was related to environmental variables that reflected a decreasing aridity gradient from left to right: variables associated to mesic conditions were positively correlated with this axis, whereas variables characterizing more xeric habitats were negatively correlated with it (Figure 4). The distribution of species along this axis identified essentially three distinct groups of species. *Anopheles arabiensis *and *An. pharoensis*, which are adapted to more arid conditions, mapped on the left of the plot. *Anopheles moucheti, An. paludis, An. ziemanni, An. nili*, *An. coustani*, and *An. hancocki*, mapped on the right side of the ordination space. *Anopheles gambiae *and *An. funestus *mapped in the central part of this axis, which--in conjunction with their high tolerance values along this axis (Table 4)--reflects their widespread distribution along this eco-climatic gradient. As previously suggested by the ENFA (Table 2), *An. arabiensis *and *An. moucheti *exhibited a narrower ecological distribution compared to other anophelines, with the lowest tolerance values observed on the first axis (Table 4). The tolerance values of *An. hancocki *and *An. pharoensis *were relatively large probably because of the limited number of presence points scattered over a number of different ecological settings (see Additional file 1), although the position of these species at both extremes of Axis 1 in Figure 4 testifies for their higher incidence in mesic and xeric conditions, respectively. Finally, the second axis, which explained only 6.7% of total variance, is more difficult to interpret, because it does not represent any unambiguous environmental gradient.

**Table 4:** Species tolerance from Canonical Correspondence Analysis

Species	Axis 1 (75.1%)	Axis 2 (6.7%)
*An. gambiae*	0.98	1.01
*An. funestus*	0.89	1.03
*An. arabiensis*	0.46	1.00
*An. nili*	0.82	0.90
*An. moucheti*	0.53	0.80
*An. paludis*	0.65	0.92
*An. ziemanni*	0.72	0.89
*An. coustani*	0.78	0.71
*An. hancocki*	1.01	0.71
*An. pharoensis*	0.92	0.78

Species tolerance as root mean of squared deviation for species through the first two axes.

## Discussion

One of the main aims of ecologists is to characterize the distribution and abundance of animal populations [57]. Knowledge of where an organism lives is a fundamental requisite for the understanding of its ecology and more detailed analyses about its biology. Nowadays, the availability of accurate environmental data over large spatial extents, together with inexpensive and powerful ways to manipulate and analyse such data, has spurred the development of analytical techniques aimed at predicting species environmental requirements, from which their geographic distribution can be inferred [58].

In tropical Africa, several studies have addressed, by different modelling approaches, the geographic distribution of malaria vectors with the objective to predict malaria transmission risk at the continental scale [12,23,59]. Most studies, however, have relied on collation of published entomological data gathered for purposes different from that of studying the vectors' geographic distribution [23,59,60]. The 'training set' of locations on which such species distribution models are based are generally a biased sample of the sites, where malaria vectors may be effectively present in nature. For example, the absence of a mosquito species in a given locality is likely to reflect the lack of medical entomologists that worked previously in that area, the nature of the collection methods, the seasonal pattern of species abundance, and other environmental or historical accidents, highlighting major limitations of distribution models relying on both accurate presence *and *absence data. At the continental scale, these limitations may be less compelling, but at higher spatial resolutions (e.g. at country-wide or regional scales) the accuracy of predictive maps based on such records could elude their intentions [59,61]. To overcome some of these limitations, this study assessed habitat suitability for malaria vectors in Cameroon with a species distribution modelling technique based only on presence data gathered from a randomized sampling plan constructed to cover all major bio-geographic domains of the country. Thus, the method used in this study fills a gap in the practical application in both the fields of spatial mapping and statistics and will serve as a stepping-stone for future comparative studies. Areas of improvement in future research will include an ability to employ other presence-only models to compare habitat predicting maps and the accuracy of the predictions [14,62].

In Cameroon, the large diversity of malaria vectors (Table 1) is non-randomly distributed across the country (Figure 2). Three different analytical approaches were employed (i.e., ENFA, discriminant analysis, and multivariate regressions) to investigate the environmental requirements and to build optimal habitat profiles of the most common malaria vectors in Cameroon [3]. One of the most significant results was the high global marginality value found for all the species concerned, indicating that these mosquitoes occupied only a specific set of environmental conditions of those available across the country. This is perhaps not surprising considering that marginality values are related to the extent of the spatial reference set, which in this case was constituted by the whole of Cameroon, a highly diversified country covering several different bio-geographic domains. Moreover, as found in other studies [32,63], eco-geographical variables (EGVs) related to human activity (distance to localities and roads) had the most important impact on the ecological niche of anthropophilic malaria vectors. These two EGVs are variables correlated to the density of roads and populated places per unit area, because they take into account the presence of spatial units occupied by localities or roads neighbouring the sampled focal unit. As such, they identify areas where anthropogenic modifications of the environment are greater. This outcome, as well as the anopheline fauna recorded in this study, could be in part related to the collection method, which focused on mosquitoes with domestic resting habits. This is a bias inherent in the fact that the sampling plan targeted those anophelines that are mostly implicated in malaria transmission, which are also those with the most anthropophilic habits (among which the behavioural trait of resting in human dwellings) [21]. *Anopheles gambiae *and *An. funestus *are highly anthropophilic and endophilic mosquitoes [33,64,65]; sampling bias is, therefore, not expected to have significantly affected the outcome of habitat suitability maps for these species, as confirmed by the excellent predictive performance of the habitat suitability models for these two species (Table 3). Conversely *An. arabiensis *is reported to have predominantly exophilic and zoophilic habits in several parts of Africa [66,67], *An. nili *and *An. moucheti *can also be highly exophilic [19,68]. Similarly to results obtained by Simard and colleagues [32], species with less anthropophilic behaviour had a weaker correlation with anthropogenic EGVs (Table 2). This difference in the strength of correlation according to degree of anthropophilic behaviour, suggests that sampling bias due to the collection technique used probably did not invalidate unduly the results of the ENFA and habitat suitability maps. It should be noted, however, that model prediction performance was lower in the case of less anthropophilic species (much so in the case of *An. moucheti*), indicating that there is still scope for improvement of the habitat suitability maps produced with the field data available in this study. Nevertheless, similar ecological species distributions were obtained by the ENFA and canonical correspondence analysis. The latter analytical technique takes into account both species presences and absences, and no EGV related to anthropogenic modifications of the environment was introduced as an explanatory variable. Thus, it can be expected that the distribution patterns observed are likely to be of general value for the less anthropophilic species too.

Despite their high global marginality, *An. gambiae *and *An. funestus *occurred in sympatry in a wide range of ecological settings (Figure 4 and Table 4). Habitat suitability maps predicted large patches of optimal habitat for both species from northern to southern Cameroon (Figure 2A, 2B). However, *An. gambiae *was comparatively more associated with conditions characterized by higher rainfall and humidity (Figure 3), which are characteristics of the equatorial rainforest, explaining why the distribution of optimal habitat in this bio-geographic domain was more extensive for this mosquito compared to *An. funestus*. It is interesting to note that both species, which showed the lowest global marginality and highest tolerance, have in Cameroon highly structured populations at the genetic level [69-71]. *Anopheles gambiae *is in fact an assemblage of populations belonging to two molecular forms. Simard and colleagues [32] analysed the ecological niche requirements of these evolutionarily diverging ecotypes, showing that when considered as separate entities, the marginality and specialization indices were more extreme than those found in the present work for *An. gambiae *considered as a single taxonomic entity. Above and beyond the subdivision of *An*. *gambiae *in molecular forms, this malaria vector exhibits also an extraordinary degree of chromosomal polymorphism, which can be related to its high capacity to adapt to a wide range of ecological conditions [72,73]. Similarly, *An. funestus *populations in Cameroon are composed of several chromosomal inversion variants with distinct geographical distributions [69]. The more 'generalist' nature of *An. gambiae *and *An. funestus *as taxonomic entities, therefore, could well result from the assemblage of natural populations of genetic variants (karyotypes) each having a specialized ecological niche [63]. Conversely, *An. arabiensis*, *An. moucheti *and *An.nili *had much more restricted and contrasting distributions of suitable habitat (Figure 2C, 2D, 2E). *Anopheles arabiensis *was mainly distributed in the most xeric habitats of northern Cameroon that are characterized by high values of evapo-transpiration and sunlight exposure (Table 2). Here, it is frequently found together with the other two major malaria vectors *An. gambiae *and *An. funestus*, contributing to high rates of parasite transmission during and soon after the rainy season in the savanna bio-geographic domain [23,61,74]. On the other hand, *An. nili *and *An. moucheti *are two 'forest' species, occurring in regions characterized by higher values of water vapour pressure and rainfall (Table 2), as is typically recorded in the equatorial rainforest of southern Cameroon. Both species, together with *An. gambiae *and, to a lower extent, *An. funestus*, sustain year-round malaria transmission in the forested regions of Cameroon [68]. Unfortunately, cytogenetic data are as yet not available for these two species to relate their ecological requirements with chromosomal polymorphism.

This study focused on the role that abiotic variables related to climate, topography, or land use have on malaria vector range and distribution, whereas biological processes such as inter-specific competition or predation were not included among the species distribution modelling predictors. Previous studies have revealed how biological interactions, particularly at the larval stage of development, can affect the population dynamics and distribution of anophelines [75,76]. For example, breeding place competition between *An. gambiae *and *An. arabiensis *can displace the former in favour of the latter [75]. Contrasting responses to aquatic predators have been considered responsible for generating differences in life-history traits between the two molecular forms of *An. gambiae *according to the nature of the breeding site [65]. The role that these interactions play in the modulation of mosquito geographic distribution and dynamics certainly warrants further research.

Several studies have reported that human disturbance of the natural environment through the action of irrigation or deforestation can favour the spread and colonization of new areas by efficient malaria vectors, increasing the risk of transmission [29,64,77,78]. In agreement with this view, large patches of optimal/suitable habitat for vectors such as *An. gambiae *or *An. funestus *occurred in densely populated areas of intensive farming and around major urban centres, whereas regions of low human population density or uninhabited areas were classified as marginal or totally unsuitable. This suggests that global environmental changes, including deforestation, urbanization, or land use conversion for agricultural purposes, as well as the ongoing demographic surge that Africa is currently experiencing are likely to impact on vector distribution and malaria epidemiology in the times to come. Future research should consider the dynamic nature of mosquito population ecology, including population genetic analyses, to understand the evolution of species ranges and current trends in malaria transmission risk.

The presence of a highly differentiated malaria vector system occurring in a given geographical area, as observed in Cameroon, can clearly have a profound impact on the nature and intensity of transmission [19,29]. In this context, fine-grained mapping of the vectors' distribution together with the identification, characterization and ranking of their ecological requirements, as well as of the ecological determinants to which mosquitoes respond, is of great interest to assess and predict disease transmission risk. Such knowledge might allow focusing vector control efforts in areas and at times where the target vector species are most amenable to control, and improve both efficacy and cost-effectiveness of disease control through vector control interventions [62,79].

## Competing interests

The authors declare that they have no competing interests.

## Authors' contributions

DA, CC, DF and FS conceived and designed the experiments. DA, GCK, CAN, JPA, PAA performed the fieldwork. DA and KO carried out the SIG analysis. DA, CC and FS analysed the data. DA drafted the manuscript, which was critically reviewed by CC and FS. All authors read and approved the final manuscript.

## Supplementary Material

Additional file 1 **Maps showing the geographic location of occurrence records of 18 anopheline species across Cameroon**. Collections were conducted inside human dwellings in 386 villages throughout Cameroon between 1998 and 2007 (Updated from Antonio-Nkondjio *et al *[22]). Asterisks indicate known malaria vectors. Click here for file

## References

[B1] Brown JH Mehlman DW Stevens GC Spatial variation in abundance Ecology 1995 76 2028 2043 10.2307/1941678

[B2] Hutchinson GE Concluding remarks Harbour Symposium on Quantitative Biology 1957 415 427

[B3] Hirzel AH Hausser J Chessel D Perrin N Ecological-niche factor analysis: How to compute habitat- suitability maps without absence data? Ecology 2002 83 2027 2036

[B4] Hirzel AH Le Lay G Helfer V Randin C Guisan A Evaluating the ability of habitat suitability models to predict species presences Ecol Model 2006 199 142 152 10.1016/j.ecolmodel.2006.05.017

[B5] Kirkpatrick M Barton NH Evolution of a species' range Am Nat 1997 150 1 23 10.1086/286054 18811273

[B6] Guisan A Thuiller W Predicting species distribution: offering more than simple habitat models Ecol Lett 2005 8 993 1009 10.1111/j.1461-0248.2005.00792.x 34517687

[B7] Cassinello J Acevedo P Hortal J Prospects for population expansion of the exotic aoudad (*Ammotragus lervia*; Bovidae) in the Iberian Peninsula: clues from habitat suitability modelling Divers Distrib 2006 12 666 678 10.1111/j.1472-4642.2006.00292.x

[B8] Pfenninger M Nowak C Reproductive isolation and ecological niche partition among larvae of the morphologically cryptic sister species *Chironomus riparius *and *C. piger* PLoS ONE 2008 3 e2157 10.1371/journal.pone.0002157 18478074 PMC2364647

[B9] Sattler T Bontadina F Hirzel AH Arlettaz R Ecological niche modelling of two cryptic bat species calls for a reassessment of their conservation status J Appl Ecology 2007 44 1188 1199 10.1111/j.1365-2664.2007.01328.x

[B10] Giovanelli J Haddad C Alexandrino J Predicting the potential distribution of the alien invasive American bullfrog (*Lithobates catesbeianus*) in Brazil Biological Invasions 2008 10 585 590 10.1007/s10530-007-9154-5

[B11] Snow RW Marsh K leSueur D The need for maps of transmission intensity to guide malaria control in Africa Parasitol Today 1996 12 455 457 10.1016/S0169-4758(96)30032-X

[B12] Rogers DJ Randolph SE Snow RW Hay SI Satellite imagery in the study and forecast of malaria Nature 2002 415 710 715 10.1038/415710a 11832960 PMC3160466

[B13] Peterson AT Ecologic niche modeling and spatial patterns of disease transmission Emerg Infect Dis 2006 12 1822 1826 17326931 10.3201/eid1212.060373 PMC3291346

[B14] Levine RS Peterson AT Benedict MQ Geographic and ecologic distributions of the *Anopheles gambiae *complex predicted using a genetic algorithm Am J Trop Med Hyg 2004 70 105 109 14993618

[B15] Peterson AT Sanchez-Cordero V Ben Beard C Ramsey JM Ecologic niche modeling and potential reservoirs for Chagas disease, Mexico Emerg Infect Dis 2002 8 662 667 12095431 10.3201/eid0807.010454 PMC2730326

[B16] Benedict MQ Levine RS Hawley WA Lounibos LP Spread of the tiger: Global risk of invasion by the mosquito Aedes albopictus Vector-Borne and Zoonotic Diseases 2007 7 76 85 10.1089/vbz.2006.0562 17417960 PMC2212601

[B17] Kleinschmidt Bagayoko M Kleinschmidt Bagayoko M Clarke GPY Craig M Sueur Dl A spatial statistical approach to malaria mapping Int J Epidemiol 2000 29 355 361 10.1093/ije/29.2.355 10817136

[B18] Kleinschmidt I Omumbo J Briet O Giesen N van de Sogoba N Mensah NK Windmeijer P Moussa M Teuscher T An empirical malaria distribution map for West Africa Trop Med Int Health 2001 6 779 786 10.1046/j.1365-3156.2001.00790.x 11679126

[B19] Fontenille D Simard F Unravelling complexities in human malaria transmission dynamics in Africa through a comprehensive knowledge of vector populations Comp Immunol Microbiol Infect Dis 2004 27 357 375 10.1016/j.cimid.2004.03.005 15225985

[B20] Hay SI Rogers DJ Toomer JF Snow RW Annual *Plasmodium falciparum *entomological inoculation rates (EIR) across Africa: literature survey, Internet access and review Trans R Soc Trop Med Hyg 2000 94 113 127 10.1016/S0035-9203(00)90246-3 10897348 PMC3204456

[B21] Mouchet J Carnevale P Coosemans M Julvez J Manguin S Richard-Lenoble D Sircoulon J Biodiversité du paludisme dans le monde 2004 Paris: John Libbey Eurotext

[B22] Antonio-Nkondjio C Kerah CH Simard F Awono-Ambene P Chouaibou M Tchuinkam T Fontenille D Complexity of the malaria vectorial system in Cameroon: contribution of secondary vectors to malaria transmission J Med Entomol 2006 43 1215 1221 10.1603/0022-2585(2006)43[1215:COTMVS]2.0.CO;2 17162956

[B23] Moffett A Shackelford N Sarkar S Malaria in Africa: Vector Species' Niche Models and Relative Risk Maps PLoS ONE 2007 2 e824 10.1371/journal.pone.0000824 17786196 PMC1950570

[B24] Hervy JF Le Goff G Geoffroy B Herve JP Manga L Brunhes J Les Anophèles de la region afrotropicale 1998 Paris, France

[B25] Brunhes J Le Goff G Bousses P Anophèles afrotropicaux. V. Description du mâle et des stades pre -imaginaux d'*An. deemingi *et description d'*An. eouzan *i n.sp. (Diptera: Culicidae) Ann Soc Entomol France 2003 39 179 185

[B26] Awono-Ambene HP Kengne P Simard F Antonio-Nkondjio C Fontenille D Description and bionomics of *Anopheles *(Cellia) *ovengensis *(Diptera: Culicidae), a new malaria vector species of the *Anopheles nili *group from south Cameroon J Med Entomol 2004 41 561 568 10.1603/0022-2585-41.4.561 15311444

[B27] Robert V Broek A van den Stevens P Slootweg R Petrarca V Coluzzi M Le Goff G Di Deco MA Carnevale P Mosquitoes and malaria transmission in irrigated rice-fields in the Benoue valley of northern Cameroon Acta Trop 1992 52 201 204 10.1016/0001-706X(92)90036-W 1363184

[B28] Fontenille D Wandji S Djouaka R Awono-Ambene HP *Anopheles hancocki*, vecteur secondaire du paludisme au Cameroun Bull Lia Doc OCEAC 2000 33 23 26

[B29] Antonio-Nkondjio C Simard F Awono-Ambene P Ngassam P Toto JC Tchuinkam T Fontenille D Malaria vectors and urbanization in the equatorial forest region of south Cameroon Trans R Soc Trop Med Hyg 2005 99 347 354 10.1016/j.trstmh.2004.07.003 15780341

[B30] Cohuet A Simard F Toto JC Kengne P Coetzee M Fontenille D Species identification within the *Anopheles funestus *group of malaria vectors in Cameroon and evidence for a new species Am J Trop Med Hyg 2003 69 200 205 13677376

[B31] Olivry JC Fleuves et Rivières du Cameroun 1986 9 Paris, France: ORSTOM

[B32] Simard F Ayala D Kamdem G Pombi M Etouna J Ose K Fotsing J-M Fontenille D Besansky N Costantini C Ecological niche partitioning between *Anopheles gambiae *molecular forms in Cameroon: the ecological side of speciation BMC Ecology 2009 9 17 10.1186/1472-6785-9-17 19460146 PMC2698860

[B33] Gillies MT de Meillon B The Anophelinae of Africa South of the Sahara 1968 54 Johannesburg: The South African Institute for Medical Research

[B34] Gillies MT Coetzee M A Supplement to the Anophelinae of Africa South of the Sahara (Afrotropical region) 1987 Johannesburg: The South African Institute for Medical Research

[B35] Scott JA Brogdon WG Collins FH Identification of single specimens of the *Anopheles gambiae *complex by the polymerase chain reaction Am J Trop Med Hyg 1993 49 520 529 8214283 10.4269/ajtmh.1993.49.520

[B36] Favia G Lanfrancotti A Spanos L Siden-Kiamos I Louis C Molecular characterization of ribosomal DNA polymorphisms discriminating among chromosomal forms of Anopheles gambiae s.s Insect Mol Biol 2001 10 19 23 10.1046/j.1365-2583.2001.00236.x 11240633

[B37] Koekemoer LL Kamau L Hunt RH Coetzee M A cocktail polymerase chain reaction assay to identify members of the *Anopheles funestus *(Diptera: Culicidae) group Am J Trop Med Hyg 2002 66 804 811 12224596 10.4269/ajtmh.2002.66.804

[B38] Kengne P Awono-Ambene P Nkondjio CA Simard F Fontenille D Molecular identification of the *Anopheles nili *group of African malaria vectors Med Vet Entomol 2003 17 67 74 10.1046/j.1365-2915.2003.00411.x 12680928

[B39] Kengne P Antonio-Nkondjio C Awono-Ambene HP Simard F Awolola TS Fontenille D Molecular differentiation of three closely related members of the mosquito species complex *Anopheles moucheti*, by mitochondrial and ribosomal DNA polymorphism Med Vet Entomol 2007 21 177 182 10.1111/j.1365-2915.2007.00681.x 17550437

[B40] Shuttle Radar Topography Mission http://www2.jpl.nasa.gov/srtm/

[B41] ESRI France Support technique http://support.esrifrance.fr/

[B42] ESRI Support Center http://support.esri.com

[B43] FAO SDdimensions http://www.fao.org/sd/2002/EN1203a_en.htm

[B44] Global Land Cover 2000 Project http://bioval.jrc.ec.europa.eu/products/glc2000/products.php

[B45] GeoNet Name Server http://earth-info.nga.mil/gns/html/index.html

[B46] Sokal RR Rohlf FJ Biometry 1981 2 New York: Freeman & Co

[B47] Hirzel AH Posse B Oggier PA Crettenand Y Glenz C Arlettaz R Ecological requirements of reintroduced species and the implications for release policy: the case of the bearded vulture J Appl Ecology 2004 41 1103 1116 10.1111/j.0021-8901.2004.00980.x

[B48] IDRISI 32-22 http://www.clarklabs.org/

[B49] Biomapper http://www.unil.ch/biomapper

[B50] Braunisch V Bollmann K Graf RF Hirzel AH Living on the edge - Modelling habitat suitability for species at the edge of their fundamental niche Ecological Modelling 2008 214 153 167 10.1016/j.ecolmodel.2008.02.001

[B51] Fielding AH Bell JF A review of methods for the assessment of prediction errors in conservation presence/absence models Environmental Conservation 1997 24 38 49 10.1017/S0376892997000088

[B52] Hirzel AH Arlettaz R Modeling habitat suitability for complex species distributions by environmental-distance geometric mean Environ Manage 2003 32 614 623 10.1007/s00267-003-0040-3 15015699

[B53] Boyce MS Vernier PR Nielsen SE Schmiegelow FKA Evaluating resource selection functions Ecol Model 2002 157 281 300 10.1016/S0304-3800(02)00200-4

[B54] Ter Braak C CANOCO - Fortran program for canconical community ordination 1987 Ithaca, NY: Microcomputer Power

[B55] Wilkes TJ Matola YG Charlwood JD *Anopheles rivulorum*, a vector of human malaria in Africa Med Vet Entomol 1996 10 108 110 10.1111/j.1365-2915.1996.tb00092.x 8834753

[B56] Akogbeto M Romano R Infectivity of *Anopheles melas *vis-a-vis *Plasmodium falciparum *in the coastal lagoon area of Benin Bull Soc Pathol Exot 1999 92 57 61 10214525

[B57] Andrewartha HG Birch LC The distribution and abundance of animals 1954 Chicago, Illinois: The University of Chicago Press

[B58] Guisan A Zimmermann NE Predictive habitat distribution models in ecology Ecol Model 2000 135 147 186 10.1016/S0304-3800(00)00354-9

[B59] Coetzee M Craig M le Sueur D Distribution of african malaria mosquitoes belonging to the *Anopheles gambiae *complex Parasitol Today 2000 16 74 77 10.1016/S0169-4758(99)01563-X 10652493

[B60] Bayoh MN Thomas CJ Lindsay SW Mapping distributions of chromosomal forms of *Anopheles gambiae *in West Africa using climate data Med Vet Entomol 2001 15 267 274 10.1046/j.0269-283x.2001.00298.x 11583443

[B61] Kiszewski A Mellinger A Spielman A Malaney P Sachs SE Sachs J A global index representing the stability of malaria transmission Am J Trop Med Hyg 2004 70 486 498 15155980

[B62] Peterson AT Shifting suitability for malaria vectors across Africa with warming climates BMC Infec Dis 2009 9 10.1186/1471-2334-9-59 PMC2694813 19426558

[B63] Costantini C Ayala D Guelbeogo W Pombi M Some C Bassole I Ose K Fotsing J-M Sagnon NF Fontenille D Besansky N Simard F Living at the edge: biogeographic patterns of habitat segregation conform to speciation by niche expansion in *Anopheles gambiae* BMC Ecology 2009 9 16 10.1186/1472-6785-9-16 19460144 PMC2702294

[B64] Coluzzi M The clay feet of the malaria giant and its African roots: hypotheses and inferences about origin spread and control of *Plasmodium falciparum* Parassitologia 1999 41 277 283 10697869

[B65] Coetzee M Fontenille D Advances in the study of *Anopheles funestus*, a major vector of malaria in Africa Insect Biochem Mol Biol 2004 34 599 605 10.1016/j.ibmb.2004.03.012 15242700

[B66] Mahande A Mosha F Mahande J Kweka E Feeding and resting behaviour of malaria vector *Anopheles arabiensis *with reference to zooprophylaxis Malar J 2007 6 100 10.1186/1475-2875-6-100 17663787 PMC1964787

[B67] Githeko AK Adungo NI Karanja DM Hawley WA Vulule JM Seroney IK Ofulla AVO Atieli FK Ondijo SO Genga IO Odada PK Situbi PA Oloo JA Some observations on the biting behavior of *Anopheles gambiae *ss, *Anopheles arabiensis*, and *Anopheles funestus *and their implications for malaria control Exp Parasitol 1996 82 306 315 10.1006/expr.1996.0038 8631382

[B68] Antonio-Nkondjio C Awono-Ambene P Toto J-C Meunier J-Y Zebaze-Kemleu S Nyambam R Wondji CS Tchuinkam T Fontenille D High malaria transmission intensity in a village close to Yaounde the capital city of Cameroon J Med Entomol 2002 39 350 355 10.1603/0022-2585-39.2.350 11931035

[B69] Cohuet A Dia I Simard F Raymond M Rousset F Antonio-Nkondjio C Awono-Ambene PH Wondji CS Fontenille D Gene flow between chromosomal forms of the malaria vector *Anopheles funestus *in Cameroon Central Africa, and its relevance in malaria fighting Genetics 2005 169 301 311 10.1534/genetics.103.025031 15677749 PMC1448888

[B70] Wondji C Simard F Fontenille D Evidence for genetic differentiation between the molecular forms M and S within the Forest chromosomal form of *Anopheles gambiae *in an area of sympatry Insect Mol Biol 2002 11 11 19 10.1046/j.0962-1075.2001.00306.x 11841498

[B71] Wondji C Frederic S Petrarca V Etang J Santolamazza F Della Torre A Fontenille D Species and populations of the *Anopheles gambiae *complex in Cameroon with special emphasis on chromosomal and molecular forms of *Anopheles gambiae *s.s J Med Entomol 2005 42 998 1005 10.1603/0022-2585(2005)042[0998:SAPOTA]2.0.CO;2 16465741

[B72] Pombi M Caputo B Simard F Di Deco MA Coluzzi M Della Torre A Costantini C Besansky NJ Petrarca V Chromosomal plasticity and evolutionary potential in the malaria vector *Anopheles gambiae *sensu stricto: insights from three decades of rare paracentric inversions BMC Evol Biol 2008 8 309 10.1186/1471-2148-8-309 19000304 PMC2654565

[B73] Coluzzi M Sabatini A della Torre A Di Deco MA Petrarca V A polytene chromosome analysis of the *Anopheles gambiae *species complex Science 2002 298 1415 10.1126/science.1077769 12364623

[B74] Antonio-Nkondjio C Ndo C Kengne P Mukwaya L Awono-Ambene P Fontenille D Simard F Population structure of the malaria vector *Anopheles moucheti *in the equatorial forest region of Africa Malar J 2008 7 120 10.1186/1475-2875-7-120 18601716 PMC2483286

[B75] Paaijmans KP Huijben S Githeko AK Takken W Competitive interactions between larvae of the malaria mosquitoes *Anopheles arabiensis *and *Anopheles gambiae *under semi-field conditions in western Kenya Acta Trop 2009 109 124 130 10.1016/j.actatropica.2008.07.010 18760989

[B76] Diabaté A Dabiré RK Heidenberger K Crawford J Lamp WO Culler LE Lehmann T Evidence for divergent selection between the molecular forms of *Anopheles gambiae*: role of predation BMC Evol Biol 2008 8 5 10.1186/1471-2148-8-5 18190719 PMC2217532

[B77] Guerra CA Snow RW Hay SI A global assessment of closed forests deforestation and malaria risk Ann Trop Med Parasitol 2006 100 189 204 10.1179/136485906X91512 16630376 PMC3204444

[B78] Vittor AY Pan W Gilman RH Tielsch J Glass G Shields T Sanchez-Lozano W Pinedo VV Salas-Cobos E Flores S Patz JA Linking deforestation to malaria in the Amazon: characterization of the breeding habitat of the principal malaria vector *Anopheles darlingi* Am J Trop Med Hyg 2009 81 5 12 19556558 PMC3757555

[B79] Tatem AJ Guerra CA Kabaria CW Noor AM Hay SI Human population urban settlement patterns and their impact on *Plasmodium falciparum *malaria endemicity Malar J 2008 7 218 10.1186/1475-2875-7-218 18954430 PMC2586635

