# Supplementary material for: Habitat suitability and ecological niche profile of major malaria vectors in Cameroon
Source: Malar J. 2009 Dec 23;8:307. doi: 10.1186/1475-2875-8-307 (PMC2805691; doi:10.1186/1475-2875-8-307)
Supplement: Additional file 1 — Maps showing the geographic location of occurrence records of 18 anopheline species across Cameroon. Collections were conducted inside human dwellings in 386 villages throughout Cameroon between 1998 and 2007 (Updated from Antonio-Nkondjio et al [22]). Asterisks indicate known malaria vectors. [file 1475-2875-8-307-S1.PDF]

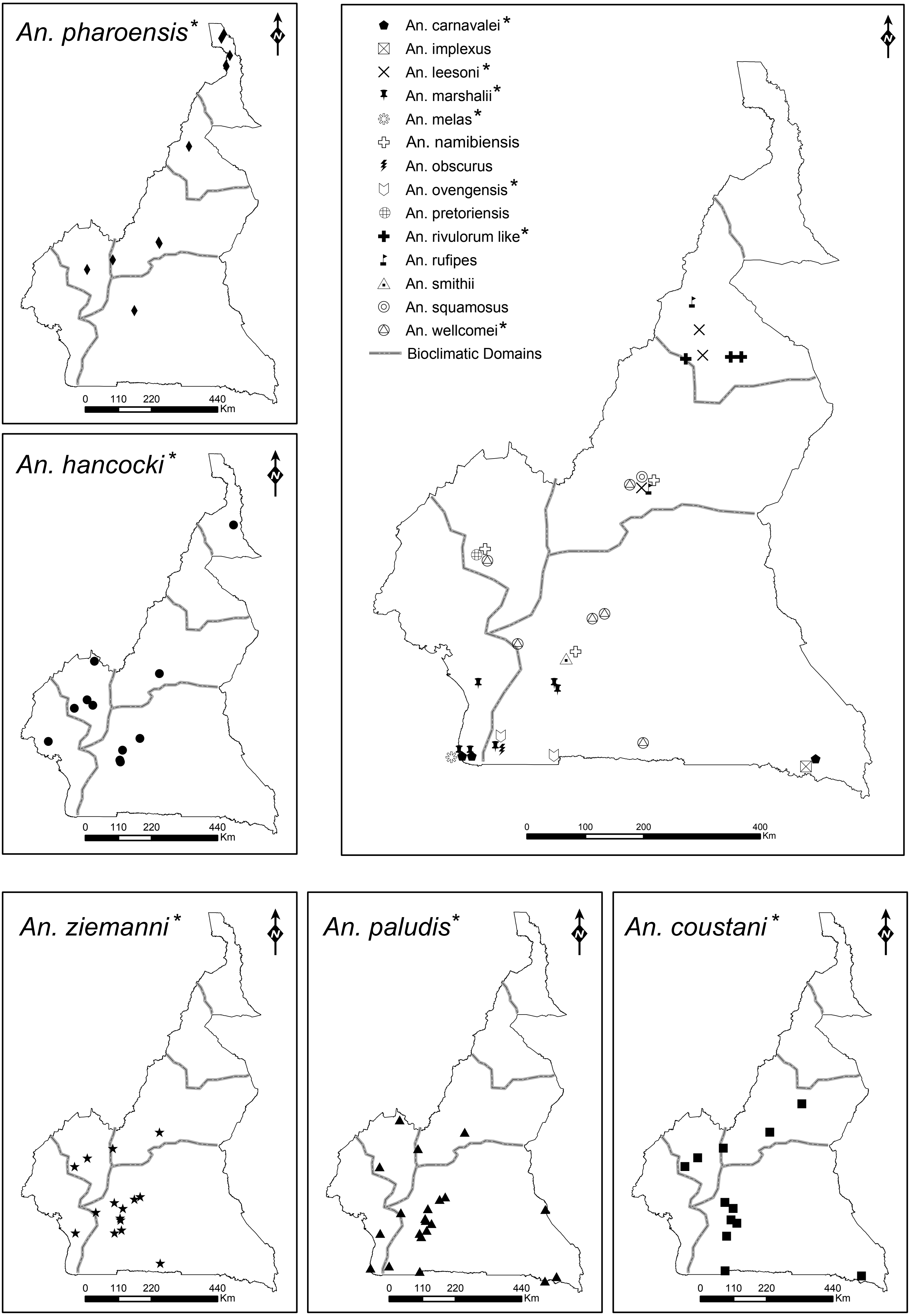
An. pharoensis*
An. hancocki*
An. carnavalei*
An. implexus
An. leesoni*
An. marshalii*
An. melas*
An. namibiensis
An. obscurus
An. ovengensis*
An. pretoriensis
An. rivulorum like*
An. rufipes
An. smithii
An. squamosus
An. wellcomei*
Bioclimatic Domains
0 100 200 400 Km
An. ziemanni*
An. paludis*
An. coustani*
0 110 220 440 Km
N
